# Supplementary material for: Deep Learning-Based Comparative Prediction and Functional Analysis of Intrinsically Disordered Regions in SARS-CoV-2
Source: Int J Mol Sci. 2025 Apr 5;26(7):3411. doi: 10.3390/ijms26073411 (PMC11989790; doi:10.3390/ijms26073411)
Supplement: Supplementary file 1 [file ijms-26-03411-s001.zip › IJMS_Supplementary Material.pdf]

Int. J. Mol. Sci. **2025**

<Supplementary Material>

Deep learning-based prediction of disordered protein regions in SARS-COV-2: a broad-spectrum antiviral drug design

Sidra Ilyas[1*], Abdul Manan[2] and Donghun Lee[1*]

[1] Department of Herbal Pharmacology, College of Korean Medicine, Gachon University, 1342 Seongnamdae-ro, Sujeong-gu, Seongnam-si, 13120, Republic of Korea; sidrailyas6@gachon.ac.kr (S.I.); dlee@gachon.ac.kr (D.L.)
[2] Department of Molecular Science and Technology, Ajou University, Suwon 16499, Korea; mananriaz012@gmail.com (A.M.)
* Correspondence: sidrailyas6@gachon.ac.kr (S.I.); dlee@gachon.ac.kr (D.L.)

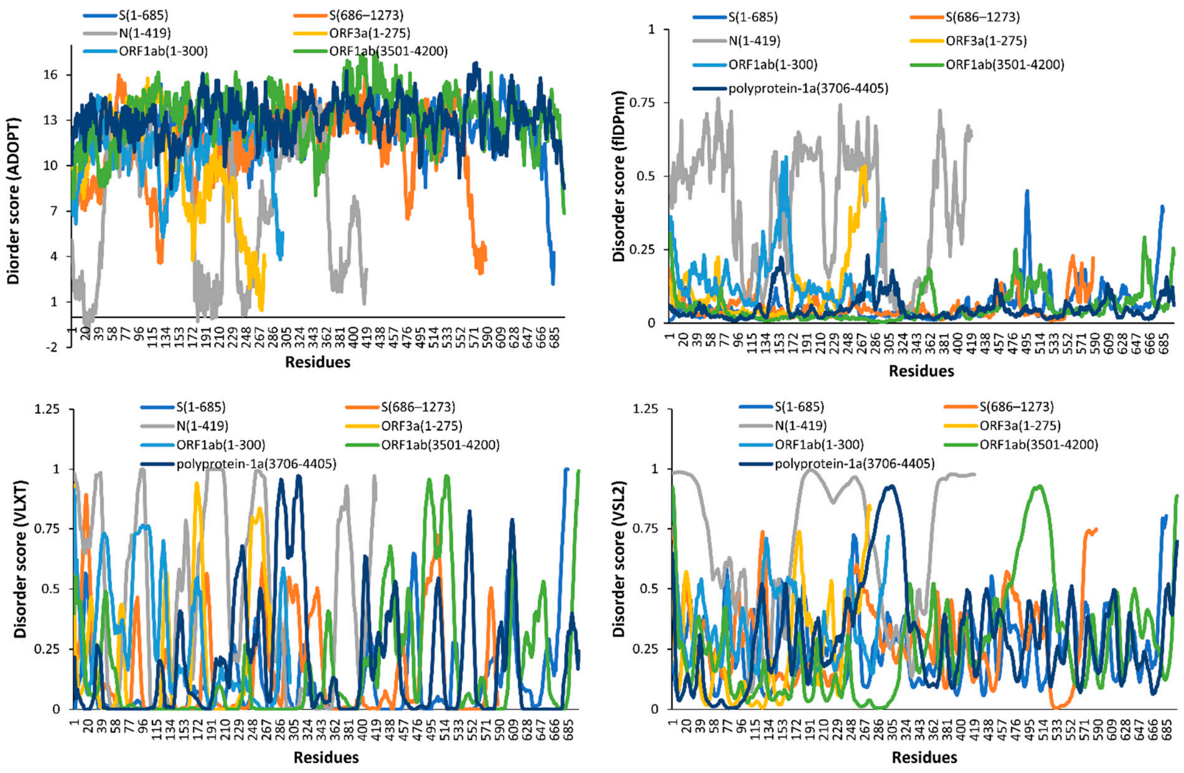

Supplementary Figure S1. Disorder propensity and Z-scores of the SARS-CoV-2 proteins predicted by ADOPT, VLXL, VSL2, and flDPnn models. ORF 1ab represents replicase polyprotein, S shows spike glycoprotein, N corresponds to nucleocapsid all of these are critical for viral function and interaction with host factors.

Supplementary sequence data S1. Protein sequences of the selected SARS-COV-2 proteins were input into ADOPT, VLXL, VSL2, and flDPnn models for IDR analysis.

>ORF1ab(1-300)

MESLVPGFNEKTHVQLSLPVLQVRDVLVRGFGDSVEEVLSEARQHLKDGTCGLVEVEKGVLPQLEQPYVF

IKRSDARTAPHGHVMVELVAELEGIQYGRSGETLGVLVPHVGEIPVAYRKVLLRKNGNKGAGGHSYGADL

KSFDLGDELGTDPYEDFQENWNTKHSSGVTRELMRELNGGAYTRYVDNNFCGPDGYPLECIKDLLARAGK

ASCTLSEQLDFIDTKRGVYCCREHEHEIAWYTERSEKSYELQTPFEIKLAKKFDTFNGECPNFVFPLNSI

IKTIQPRVEKKKLDGFMGRI

>ORF1ab(3501-4200)

NYEPLTQDHVDILGPLSAQTGIAVLDMCASLKELLQNGMNGRTILGSALLEDEFTPFDVVRQCSGVTFQS

AVKRTIKGTHHWLLLTILTSLLVLVQSTQWSLFFFLYENAFLPFAMGIIAMSAFAMMFVKHKHAFLCLFL

LPSLATVAYFNMVYMPASWVMRIMTWLDMVDTSLSGFKLKDCVMYASAVVLLILMTARTVYDDGARRVWTLMN

VLTLVYKVYYGNALDQAISMWALIISVTSNYSGVVTTVMFLARGIVFMCVEYCPIFFITGNTLQCIM

LVYCFLGYFCTCYFGLFCLLNRYFRLTLGVYDYLVSTQEFRYMNSQGLLPPKNSIDAFKLNIKLLGVGGK

PCIKVATVQSKMSDVKCTSVVLLSVLQQLRVESSSKLWAQCVQLHNDILLAKDTTEAFEKMVSLLSVLLS

MQGAVDINKLCEEMLDNRATLQAIASEFSSLPSYAAFATAQEAYEQAVANGDSEVVLKKLKKSLNVAKSE

FDRDAAMQRKLEKMADQAMTQMYKQARSEDKRAKVTSAMQTMLFTMLRKLDNDALNNIINNARDGCVPLNIIP

LTTAAKLMVVIPDYNTYKNTCDGTTFTYASALWEIQQVVDADSKIVQLSEISMDNSPNLAWPLIVTA

LRANSAVKLQNNELSPVALRQMSCAAGTTQTACTDDNALAYYNTTKGGRFVLALLSDLQDLKWARFPKSD

>polyprotein-1a(3706-4405)

RRVWTLMNVLTLVYKVYYGNALDQAISMWALIISVTSNYSGVVTTVMFLARGIVFMCVEYCPIFFITGNTLQCIMLV

YCFLGYFCTCYFGLFCLLNRYFRLTLGVYDYLVSTQEFRYMNSQGLLPPKNSIDAFKLNIKLLGVGGKPCIKVATVQS

KMSDVKCTSVVLLSVLQQLRVESSSKLWAQCVQLHNDILLAKDTTEAFEKMVSLLSVLLSMQGAVDINKLCEEML

DNRATLQAIASEFSSLPSYAAFATAQEAYEQAVANGDSEVVLKKLKKSLNVAKSEFDRDAAMQRKLEKMADQAM

TQMYKQARSEDKRAKVTSAMQTMLFTMLRKLDNDALNNIINNARDGCVPLNIIPLTTAAKLMVVIPDYNTYKNT

CDGTTFTYASALWEIQQVVDADSKIVQLSEISMDNSPNLAWPLIVTALRANSAVKLQNNELSPVALRQMSCAAGTT

QTACTDDNALAYYNTTKGGRFVLALLSDLQDLKWARFPKSDGTGTIYTELEPPCRFVTDTPKGPKVKYLYFIKGLN

NLNRGMVLGSLAATVRLQAGNATEVPANSTVLSFCAFAVDAAKAYKDYLASGGQPITNCVKMLCTHTGTGQAIT

VTPEANMDQESFGGASCCLYCRCHIDHPNPKGFCDLKGKYVQIPTTCANDPVGFTLKNTVCTVCGMWKGYGCSC

DQLREPMLQSADAQSFLNGFAV

>Spike (1-685)

MFVFLVLLPLVSSQCVNLTTRTQLPPAYTNSFTRGVYYPDKVFRSSVLHSTQDLFLPFFSNVTWFHAIHV

SGTNGTKRFDNPVLPFNDGVYFASTEKSNIIRGWIFGTTLDSKTQSLLIVNNATNVVIKVCEFQFCNDPF

LGVYYHKNNKSWMESEFRVYSSANNCTFEYVSQPFLMDLEGKQGNFKNLREFVFKNIDGYFKIYSKHTPI

NLVRDLPQGFSALEPLVDLPIGINITRFQTLLALHRSYLTPGDSSSGWTAGAAAYYVGYLQPRTFLLKYN

ENGTITDAVDCALDPLSETKCTLKSFTVEKGIYQTSNFRVQPTESIVRFPNITNLCPFGEVFNATRFASV

YAWNRKRISNCVADYSVLYNSASFSTFKCYGVSPTKLNDLCFTNVYADSFVIRGDEVRQIAPGQTGKIAD

YNYKLPDDFTGCVIAWNSNNLDSKVGGNYNYLYRLFRKSNLKPFERDISTEIYQAGSTPCNGVEGFNCYF

PLQSYGFQPTNGVGYQPYRVVVLSFELLHAPATVCGPKKSTNLVKNKCVNFNFNGLTGTGVLTESNKKFL

PFQQFGRDIADTTDAVRDPQTLEILDITPCSFGGVSVITPGTNTSNQVAVLYQDVNCTEVPVAIHADQLT

PTWRVYSTGSNVFQTRAGCLIGAEHVNNSYECDIPIGAGICASYQTQTNSPRRAR

>Spike (686–1273)

SVASQSIIAYTMSLGAENSVAYSNNSIAIPTNFTISVTTEILPVSMTKTSVDCTMYICGDSTECSNLLLQYGSFCTQLNR

ALTGIAVEQDKNTQEVFAQVKQIYKTPPIKDFGGFNFSQILPDPSKPSKRSFIEDLLFNKVTLADAGFIKQYGDCLGDI

AARDLICAQKFNGLTVLPPLLTDEMIAQYTSALLAGTITSGWTFGAGAALQIPFAMQMAYRFNGIGVTQNVLYEN

QKLIANQFNSAIGKIQDSLSSTASALGKLQDVVNQNAQALNTLVKQLSSNFGAISSVLNDILSRLDKVEAEVQIDRLI

TGRLQSLQTYVTQQLIRAAEIRASANLAATKMSECVLGQSKRVDFCGKGYHLMSFPQSAPHGVVFLHVTYVPAQE

KNFTTAPAICHDGKAHFPREGVFVSNGTHWFVTQRNFYEPQIITTDNTFVSGNCDVVIGIVNNTVYDPLQPELDSFK

EELDKYFKNHTSPDVDLGDISGINASVVNIQKEIDRLNEVAKNLNESLIDLQELGKYEQYIKWPWYIWLGFIAGLIAI

VMVTIMLCCMTSCCSCLKGCCSCGSCCKFDEDDSEPVLKGVKLHYT

>N_protein(1-419)

MSDNGPQNQRNAPRITFGGPSDSTGSNQNGERSGARSKQRRPQGLPNNTASWFTALTQHGKEDLKFPRGQGVPIN

TNSSPDDQIGYYRRATRRIRGGDGKMKDLSPRWYFYYLGTGPEAGLPYGANKDGIIWVATEGALN

TPKDHIGTRNPANNAAIVLQLPQGTTLPKGFYAEGSRGGSQASSRSSSRSRNSSRNSTPGSSRGTSPARM

AGNGGDAALALLLLDRLNQLESKMSGKGQQQQGQTVTKKSAAEASKKPRQKRTATKAYNVTQAFGRRGPE

QTQGNFGDQELIRQGTDYKHWPQIAQFAPSASAFFGMSRIGMEVTPSGTWLTYTGAIKLDDKDPNFKDQV

ILLNKHIDAYKTFPPTEPKKDKKKKADETQALPQRQKKQQTVTLLPAADLDDFSKQLQQSMSSADSTQA

>ORF3a(1-275)

MDLFMRIFTIGTVTLKQGEIKDATPSDFVRATATIPIQASLPFGWLIVGVALLAVFQSASKIITLKKRWQ

LALSKGVHFVCNLLLLFVTVYSHLLLVAAGLEAPFLYLYALVYFLQSINFVRIIMRLWLCWKCRSKNPLL

YDANYFLCWHTNCYDYCIPYNSVTSSIVITSGDGTTSPISEHDYQIGGYTEKWESGVKDCVVLHSYFTSD

YYQLYSTQLSTDTGVEHVTFFIYNKIVDEPEEHVQIHTIDGSSGVVNPVMEPIYDEPTTTTSVPL

Supplementary Table S1: Comparative analysis of experimental disordered regions validations with the predicted disorder regions by models (ADOPT, flDPnn, VLXT, and VSL2).

Proteins	Experiment	ADOPT	flDPnn	VLXT	VSL2
ORF 1ab(1-300)	129-147	2-8	156,158,163	1-6	1-8
	163-167	126-136		35-51	38-42
		288-289		82-110	78
		291-300		121-128	126-140
				169-173	147
				288-293	150-151
					159-168
					170-171
					245-250
					290
					293-300
ORF 1ab(3501-4200)	3660-3681	3503		3502-3504	3501-3506
	3982-4007	4198-4200		3930-3943	3830-3832
				3964	3863-3864
				3985-4026	3965-4030
				4107-4113	4141
				4150-4152	4195-4200
				4192-4200	
Polyprotein 1a	4393-4405			3929-3943	3706-3708
				3964	3829-3831
				3985-4026	3862-3863
				4107-4113	3965-4030
				4150-4152	4141
				4208-4211	4259, 4391,4393
				4249-4258	4401-4405
				4309-4318	
ORF 3a(1-275)	1 - 41	18-19	267-278	1-2	1
		160-181	272	127-130	18-23
		184-189		167-180	169-183

		195,197,221		244-267	221-222
		223-225			263-275
		233, 235-			
		275			
					530
					676-685
Spike (686-1275)	686	686-688		686	687-691
	1157-1162	695		697-709	703-704
	1172-1177	699-701		869-871	806-815
	1194-1198	703		945-950	934-950
		724-726		982-986	1145-1156
		793-801		992-994	1255-1276
		803		1023	
		805-820		1174-1194	
		862-863		1264	
		1161-1165			
		1167-1168			
		1249-1252			
		1254-1276			
N-protein	1 - 68	1-47	1,7,11-12	1-43	1-84
	171-248	98	14	73-102	93-102
	362 - 419	172-216	16-19	145-149	142-143
		231	21-22	152-160	145-149
		234-268	24-25	170	163-289
		270	32	172-215	355-419
		273-286	35-57	235-271	
		365-419	59-75	365-387	
			77-88		
			143-147		
			149		
			172-211		
			213,235		
			237-267		
			269		
			275-277		
			279-289		
			368, 371		
			373-383		
			397		
			411-149		

Supplementary Table S2: Statistical analysis of disorder prediction models using the Chi-Squared test.

Chi-squared test	Correlation (r)	p-value
flDPnn	4.4580	0.0347
VLXT	7.7557	0.0054
VSL2	46.8205	<0.0000
ADOPT	13.0888	0.0003
